# Lirioresinol B dimethyl ether inhibits NF-κB and COX-2 and activates IκBα expression in CCl_4_-induced hepatic fibrosis

**DOI:** 10.1186/s12906-020-2839-3

**Published:** 2020-02-11

**Authors:** Adeeb Shehzad, Shagufta Rehmat, Salman Ul-Islam, Rizwan Ahmad, Meneerah Aljafary, Noor A. Alrushaid, Ebtesam A. Al-Suhaimi

**Affiliations:** 1 Department of Clinical Pharmacy, Institute for Research and Medical Consultations, Imam Abdulrahman Bin Faisal University, Dammam, Saudi Arabia; 2 Department of Biomedical Engineering and Sciences, School of Mechanical and Manufacturing Engineering (SMME), National University of Sciences and Technology, Islamabad, Pakistan; 3 School of Life Sciences, Kyungpook National University, Daegu, South Korea; 4 Natural Products and Alternative Medicines, College of Clinical Pharmacy, Imam Abdulrahman Bin Faisal University, Dammam, Saudi Arabia; 5 Department of Biology, College of Sciences, Imam Abdulrahman Bin Faisal University, Dammam, Saudi Arabia; 6 Institute for Research and Medical Consultations, Imam Abdulrahman Bin Faisal University, Dammam, Saudi Arabia

**Keywords:** Inflammation, Lirioresinol B dimethyl ether, Antioxidant, NFκB, COX-2, Hepatic fibrosis

## Abstract

**Background:**

Inflammation is one of the key components in the initiation and progression of hepatic diseases. If not treated, inflammation may cause cell dysplasia, and ultimately cancer. In the current study, we investigated the anti-inflammatory and anti-cancer activities of plant isolated compound Lirioresinol B Dimethyl Ether (LBDE) extracted from the seeds of *Magnolia fargesii* CHENG (Magnoliaceae) against HepG2 cells as well as in BALB/C male mice.

**Methods:**

We assessed the antioxidant and anti-proliferative effects of plant compounds using DPPH assay and HepG2 cell lines. Carbon tetrachloride (CCl_4_) and Diethylnitrosamine (DEN) were used to induce liver cell dysplasia followed by hepatocellular carcinoma (HCC) in BALB/C male mice for 12 weeks. We investigated the underlying mechanism by using histopathology and immunoblot experiments.

**Results:**

Intraperitoneal injection of LBDE (50 mg/kg body weight/day) inhibited CCl_4_-induced HCC. Free radical scavenging assay shows the strong anti-oxidant activity of LBDE. Western blot results show that LBDE down-regulated nuclear factor kappa B (NFκB) and cyclooxygenase (COX-2) by preventing the phosphorylation of I kappa B alpha (IκBα) in CCl_4_ treated group. LBDE also improved liver function by decreasing Alkaline Phosphatase (ALP), aspartate aminotransferase (AST) and Alanine Aminotransferase (ALT) levels. Histopathology results revealed that LBDE decreased granulomas and express normal morphology of hepatocytes.

**Conclusions:**

These preliminary results show that LBDE has the potential to inhibit CCl_4_-induced liver cell dysplasia and prevents cancer development by regulating NFκB/COX-2 activation.

## Background

Inflammation is a complex physiological process, occurs upon exposure of the body to deleterious stimuli and conditions, such as infection and tissue injury [1]. Chronic inflammation causes the development of various diseases by recruiting inflammatory cytokines and transcription factors [2]. There is compelling evidence that inflammation promotes the development of various cancers through abnormal gene regulation and signal transduction [1, 2]. Among various cancers, hepatocellular carcinoma (HCC) is characterized by inflammatory lesions in liver cells resulting in liver dysfunction and formation of fibrosis [1]. According to estimates of the American Cancer Society, a total of 42,220 new cases (30,610 in men and 11,610 in women) are expected to diagnose and a total of 30,200 people (20,540 men and 9660 women) are expected to die from liver cancer in 2018, respectively [3]. HCC is now regarded as the fourth leading cause of cancer-related deaths in men and the eighth leading cause of deaths in women in the USA [3]. It has been noted that the incidence of HCC is alarming in Asia and Africa, where excessive alcoholism and hepatitis B and C are among the most common risks for chronic liver disease and HCC [4]. Liver is the main detoxification organ of the body responsible for the metabolism of therapeutic drugs and the elimination of toxic chemicals [5]. It is expected that HCC cases will continue to increase in the coming years; therefore, new therapeutic strategies are urgently required to control inflammatory liver diseases particularly HCC.

Liver inflammation is a progressive disorder involving the recruitment of various proinflammatory molecules such as interleukins, cytokines, and nuclear-factor kappa B (NF-κB) [6, 7]. Several studies have implicated the activation of transcription factor NF-κB in various inflammation-induced cancers including HCC [8]. Upon activation, NF-κB is translocated from cytoplasm to the nucleus, where it encodes and activates many downstream targets, including inducible nitric oxide synthase (iNOS) and cyclooxygenase-2 (COX-2), which are inflammatory mediators and involved in the development of various cancers including HCC [7, 9]. NF-κB activation increases cell proliferation, inhibit apoptosis as well as induces invasion and metastasis of cancer cells [10]. In various cancers, NF-κB is activated due to inflammation in the tumor microenvironment or mutation in the upstream components of NF-κB signaling pathway [8, 10]. Thus, NF-κB is playing a critical role in mediating the inflammation-induced cancers. Additionally, COX-2 overexpression has been reported in chronic hepatitis, cirrhosis and carcinogen-induced HCC models. COX-2-induced prostaglandin E_2_ (PGE_2_) productions mediate cellular proliferation and provoke inflammatory processes in various tumorigenic models. Increased expression of PGE_2_ has been reported in the cancerous hepatocytes as well as involved in tumor progression, angiogenesis, proliferation, and survival [11]. Therefore, chronic expression of both COX-2 and NF-κB mediate propagation of hepatic inflammation as well as involved in the development of HCC. Hence, inhibition of NF-κB and COX-2 will be a better therapeutic strategy in the treatment of chronic hepatitis and HCC.

**Fig. 1:**
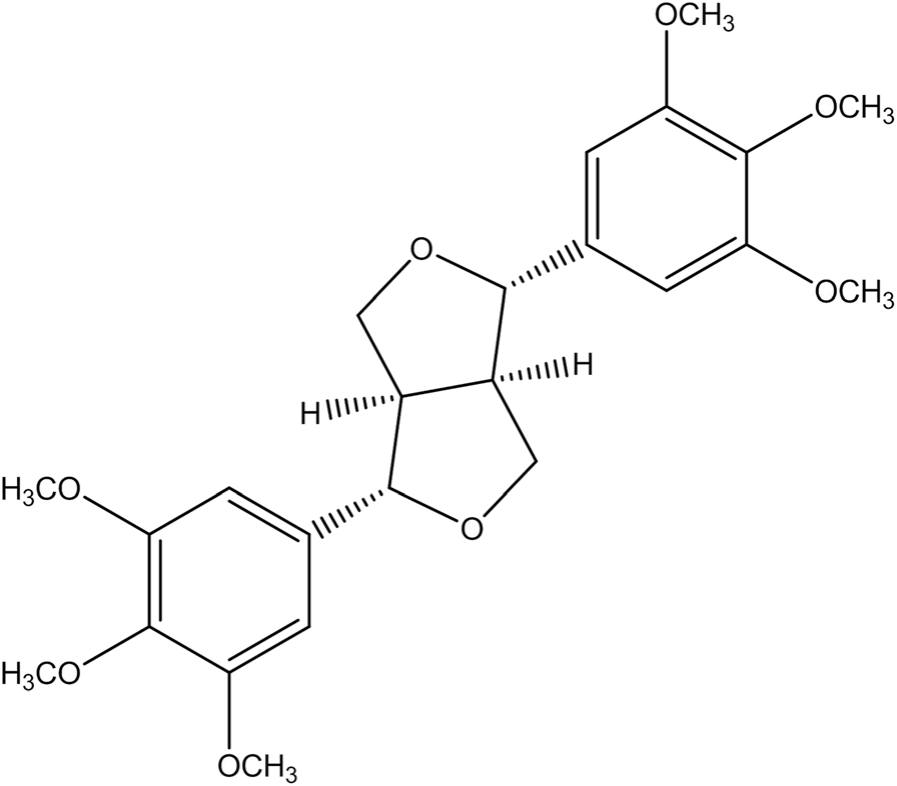
Chemical structure of LBDE

Lirioresinol B Dimethyl Ether (LBDE) is tetrahydrofurofuran-type lignin obtained from the seeds of *Magnolia fargesii* CHENG (Magnoliaceae) (https://wcsp.science.kew.org/namedetail. do?name_id=117568), which possesses a wide range of pharmacological properties. This phytochemical has been found to possess 6 methoxyl groups, 8 aliphatic protons and 4 aromatic protons [Fig. 1]. Previous studies have reported the antimicrobial, anti-depressant, anti-rhinitis and anti-inflammatory effects of LBDE [12]. Oral administration of MF extract reduced ovariectomy-induced bone loss by inhibiting the matrix metalloproteinases and receptor activator of nuclear factor-κB ligand (RANKL)-mediated osteoporosis progression [13]. LBDE has been reported to suppress breast cancer metastases and bone microenvironment by inhibiting the parathyroid hormone-related protein (PTHrP), matrix metallopeptidase 9 (MMP-9) and cathepsin K activities in breast cancer cells and osteoclastic bone resorption [14].

CCl_4_ is a renowned hepatotoxic chemical, which causes hepatic injuries in the experimental models through activation of COX-2 and NF-κB inflammatory signalling pathways [15, 16]. Therefore, inhibition of these inflammatory signalling could open a therapeutic window for anti-inflammatory and hepatoprotective agents against hepatic diseases. Our present study was designed to investigate the underlying mechanism of LBDE against CCl_4_-induced hepatic fibrosis and its relationship with activation of COX-2 and NF-κB in in-vitro and in-vivo.

## Methods

### Chemicals and reagents

Diethylnitrosamine (DEN) and CCl_4_ were purchased from Sigma Chemical Co. (St. Louis, MO, USA). *Magnolia fargesii* with a voucher specimen (Lee, J. & M. S. Yang 021) was obtained from Gyeongsang National University, South Korea. LBDE with molecular weight 446.5 g/mol was isolated as described previously [17]. Polyclonal rabbit anti-rat NF-κBP65 and I-κBα were purchased from Santa Cruz Biotechnology (Santa Cruz, CA). Anti-rat secondary antibody labelled with horseradish peroxidase was taken from Cell Signalling (Beverly, MA). The Nitrocellulose membrane was used for the transfer proteins (Bio-Rad, CA). The visualisation of proteins was done with ECL and detection kit (Amersham Pharmacia Biotech, UK). A bicinchonic acid (BCA) protein assay kit for protein quantification was provided by Pierce Biotechnology, Rockford.

### Free radical scavenging activity

Free radical scavenging activity of LBDE was done to measure its antioxidant activity by using 1, 1-Diphenyl-2-picryl-hydrazyl (DPPH) assay. This assay was started by making a stock solution of DPPH of 10 mM and was kept in dark. To compare antioxidant activity of LBDE, four different concentrations of solutions were prepared along with 0.1 mM DPPH. The reaction took place after 30 to 35 min, with a change in colour from dark purple to light yellow. This reaction was observed under UV-VIS spectrophotometer (UV-2800) at 517 nm. Absorbance values at the time of reaction were noted and used to calculate percentage anti-oxidant activity (%AA) as previously described [18, 19].

$$ AA\left(\%\right)=\frac{\left( Abs\  of\ control\right)-\left( Abs\  of\ sample\right)\times 100}{Abs\  of\ control} $$

### Cell culture and cell treatments

Human hepatoma (HepG2) and Human embryonic kidney (HEK-293) cell lines (ATCC, Rockville, MD, USA), was cultured in the Dulbecco’s modified Eagle’s medium (DMEM; Gibco, ThermoFisher Scientific, NY, USA) containing 10% fetal bovine serum (FBS). Cells were grown in a 5% CO_2_ atmosphere at 37 °C. LBDE was dissolved in the dimethyl sulfoxide (DMSO; Sigma, St. Louis, MO, USA) with a final concentration of 0.1% in the medium and cells were exposed to different concentrations (5, 10, 20, 40, and 80 μM) of LBDE for 24 h. Control cells were only treated with 0.1% DMSO.

### MTT cell viability assay

To measure the viability of HepG2 cells, cells were seeded at a density of 1 × 10^6^ cells/mL in a 96-well microtiter plate and then exposed to various concentrations of LBDE. Cells treated with DMSO served as a control. Cells were exposed to various concentrations (5, 10, 20, 40, and 80 μM) of LBDE for 24 h. Then, 3-(4,5-dimethylthiazol-2-yl)-2,5-diphenyltetrazolium bromide (MTT) was dissolved in 1X PBS (5 mg/mL) and total of 50 μL was added to each well. After 2 h incubation at 37 °C, total of 100 μL DMSO (Sigma) was added to dissolve the crystals in each well. Finally, it was gently agitated and read at 570 nm using a spectrophotometer (Bio-Rad, CA). Non-viable cells are unable to reduce the MTT dye, giving an indirect measure of LBDE effects on cell number, where control cells were considered as 100% viable. The concentration of LBDE that reduced cell proliferation by 50% (IC50) was calculated by non-linear regression fit using GraphPad Prism. The selectivity index (SI) was also calculated from the IC50 ratio of HEK-293 and HePG2 cells as previously shown [20, 21]. SI value indicates the selectivity of the LBDE against HEK-293 and HePG2 cell lines tested.

### Animal study

BALB/C male mice were purchased from the National Institute of Health, Islamabad, Pakistan. The mice were examined and housed in metal cages in a 12/12 h  day/night cycle photoperiod. The experimental protocol was approved by the Institutional Review Board and Ethics Committee *IRB/SMME/NUST/12–3/2018*, and agrees with international laws and policies (NIH Guide for the Care and Use of Laboratory Animals, NIH publication No. 85–23, 1985). A total of 20 male mice of 2–3 weeks old were categorized into three groups, 6 mice in each group based on weighing each one. Groups were labelled as Control, CCl_4_, and CCl_4_ + LBDE. All of them were placed in the same environmental conditions and were kept for a week to acclimatize. A mouse was weighed and was noted for each. These weights were then averaged to calculate the dose of CCl_4_, DEN, and LBDE.

### Animal treatment

Liver inflammation followed by HCC was induced by intraperitoneal (i.p) injection of 10% of CCl_4_ at a dose of 0.5 mL/kg of body weight dissolved in olive oil and was administered on alternate days of the week for twelve consecutive weeks [16]. DEN was used as a promoter to enhance the effect of CCl_4_. For this purpose, 1% DEN diluted in normal saline at a dose of 50 mg/kg was administered orally by gavage once a week for twelve consecutive weeks [22]. The control groups received normal saline via the same route. The BALB/C male was separated into three groups (= 6): (A) the control group, which received just distilled water; (B) the CCl_4_ group and DEN-treated group; (C) the DEN+CCl_4_ + LBDE group. LBDE was dissolved in the DMSO and a total dose of 50 mg/kg was given intraperitoneally according to weight of the mice for 4 weeks, thrice a week. The doses were selected based on our preliminary study (unpublished data), and comparable doses in previous reports, adjusting for the difference in the route of application [23]. This treatment was given only to the third experimental group labelled as CCl_4_ + LBDE. Treatment injections were administered on alternate days of the week. After induction of disease and treatment with LBDE, blood was collected before mice were sacrificed by cervical dislocation and liver tissue samples were collected.

### Histological analysis

Liver fragments were fixed in 10% formalin for 24 h, before being processed and embedded in paraffin. Five sections of 4–5 m from each group were cut and mounted on glass slides. The slices were stained with hematoxylin-eosin and examined by an inverted microscope (Observer Z1, Zeiss MicroImaging, GmbH) equipped with a camera and 4.7.4 image analysis software (AxionCam MRm Zeiss) at a magnification of 400x [24].

### Liver function tests (LFTs)

Liver function tests were carried out from blood samples of experimental models (mice). Alkaline phosphatase (ALP), aspartate aminotransferase (AST) and alanine aminotransferase (ALT) tests were done at the Diagnostic lab of Atta-Ur-Rahman School of Applied Biosciences by using Microlab-300 (Eli Tech Group, Netherlands). Serum and liver tissue samples were collected at the end of the 12-week treatment. The liver/body weight (BW) ratio was calculated according to the formula as previously reported [25].

### Protein isolation and SDS electrophoresis

Fresh liver was dissected and stored in the liquid nitrogen and then was homogenized with 1 mL of tissue lysis buffer containing 20 mM Tris, 137 mM NaCl, and 10% glycerol, with protease inhibitors. The cell lysates were centrifuged at 7000×g for 20 min, and supernatant fraction was stored in a separate in a tube. The protein concentration was quantified using BCA protein assay. Aliquots of 20 μg were diluted with 4X loading buffer, boiled for 5 min and run immediately on 10 or 12% acrylamide SDS–PAGE gels as described previously [26]. Proteins were then separated by SDS-PAGE and transferred onto nitrocellulose membranes using transfer buffer. The blots were blocked with 5% non-fat milk in Tris buffer saline containing 0.1% Tween 20 (1x TBST) and then incubated with primary antibodies primary antibody to each specific protein followed by secondary antibodies. Proteins were transferred electrophoretically to nitrocellulose membranes, and the membranes were blocked in 5% skim milk/Tris-buffered saline and reacted with primary antibody to each specific protein and visualized using ECL reagents as described previously [27].

### Statistical analysis

All results are expressed as mean values ± standard deviation. Data were analysed by Image J software (Microsoft Java 1.1.4). Difference and comparison between the groups were determined using the Student’s t-test and one-way analysis of variance (ANOVA).

## Results

### Anti-oxidant activity of LBDE

**Fig. 2:**
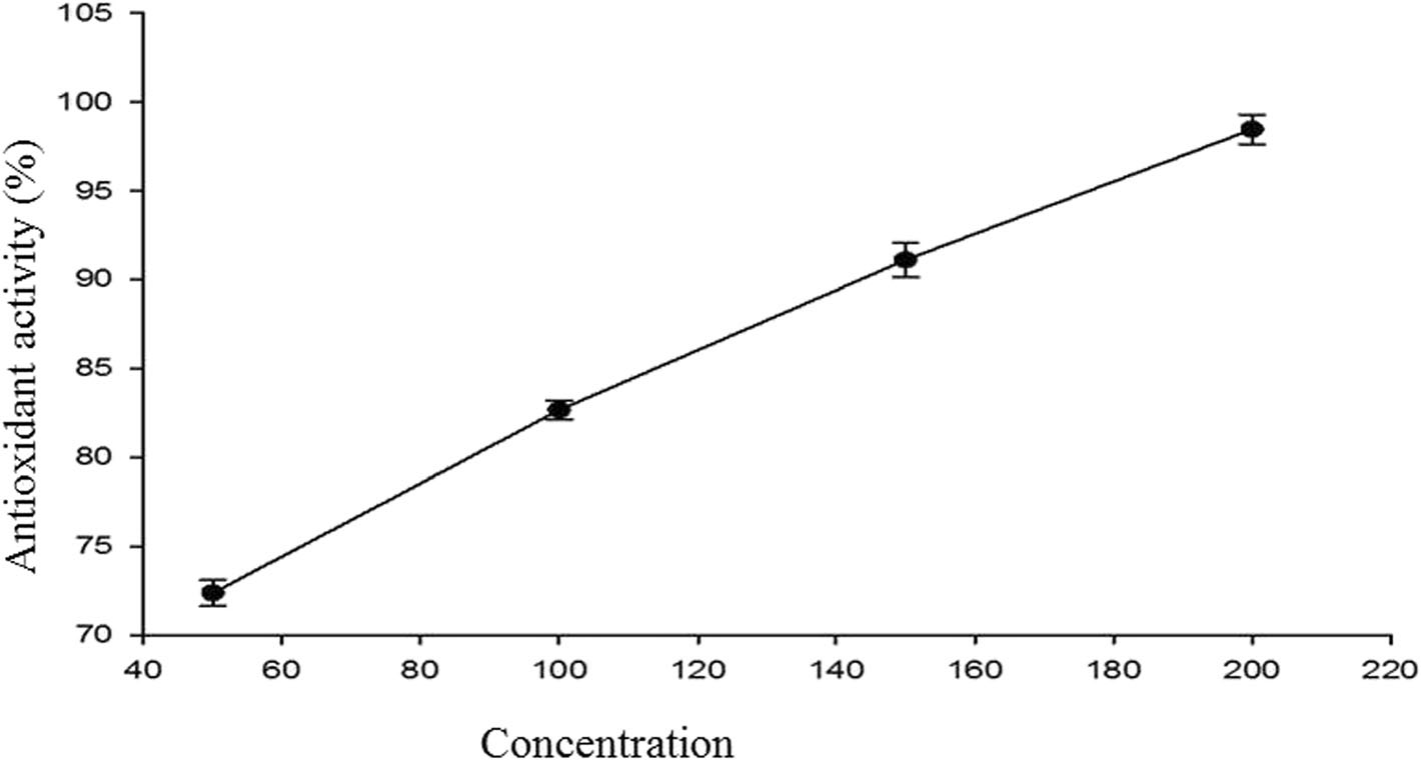
Percentage anti-oxidant activity (%AA) of LBDE at different concentrations

LBDE was evaluated for its anti-oxidant activity by DPPH assay. Four different concentrations of LBDE were compared for antioxidant activity. Three readings were obtained against each concentration and results showed that anti-oxidant activity was increased with an increase in concentration, as shown in Fig. 2. This figure shows that percentage anti-oxidant activity (%AA) with an IC50 value of 110.9 μM.

### LBDE inhibits HepG2 cells viability

**Fig. 3:**
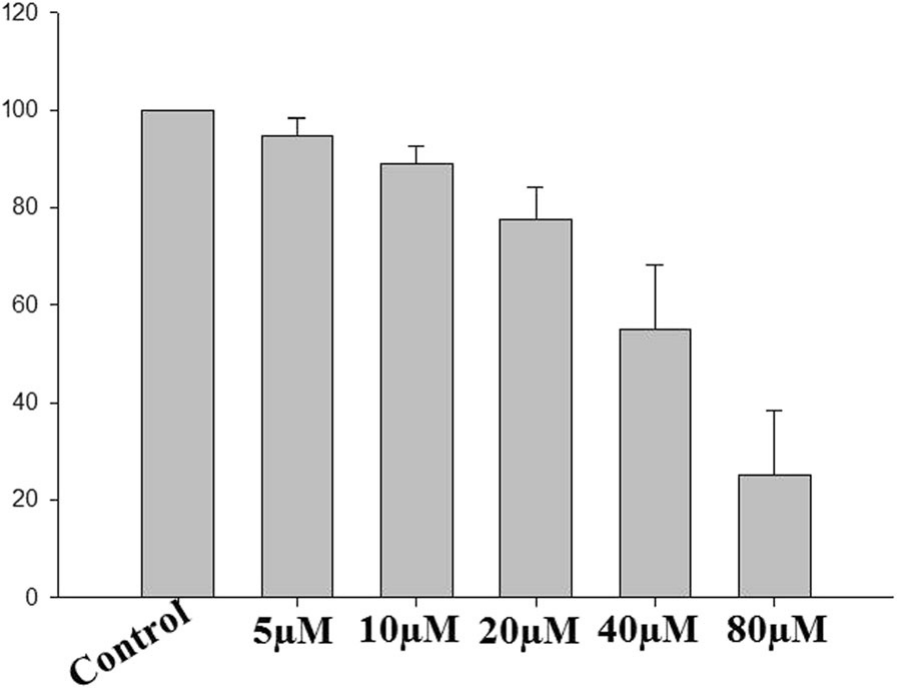
HepG2 cells were treated with increasing concentration of LBDE and analysed by MTT assay. Data represent the average ± SD of three independent experiments for each bar. *P* ≤ 0.05

To confirm the potential of LBDE on cells viability, HepG2 cells were exposed to various concentration of LBDE for 24 h and cells viability was determined by MTT assay. As shown in Fig. 3, the viability of HepG2 cells was decreased with the treatment of LBDE in a dose-dependent manner with an IC50 value of 27.97 μM. It is also confirmed that the inhibitory effect of LBDE was not due to cytotoxicity, but an anti-inflammatory effect on HepG2 cell lines, as observed with SI values. The SI value for LBDE was noted 1.07, which is indicative of the anti-inflammatory effect.

### LBDE treatment alleviated hepatic damage

During the experiments, no deaths occurred in the control and LBDE treated group, whereas one mouse died in the CCl_4_-treated group. As shown in Table 1, the body weight regained considerably upon treatment with LBDE as compared with the CCl_4_-treated group. Also, liver weight and the liver index have been improved after treatment with LBDE as compared to normal group. As shown in Table 1, LBDE treatment significantly decreased liver weight and index as compared to the CCl_4_ group.

**Table 1:** Effects of LBDE on body weight, liver weight and liver index of BALB/C

Group	No	Body weight (g)	Liver weight (g)	Liver index (Liver weight/Body weight)
Control	6	35.1 ± 2.5	5.49 ± 0.07	0.156 ± 0.003
CCL4	5	27.6 ± 2.9	7.85 ± 0.09	0.284 ± 0.006
CCL4 + LBDE(50 mg/kg)	6	31.3 ± 3.7.	6.37 ± 0.26	0.203 ± 0.002

Each value represents the mean ± SD

### LBDE treatment improved liver function

**Fig. 4:**
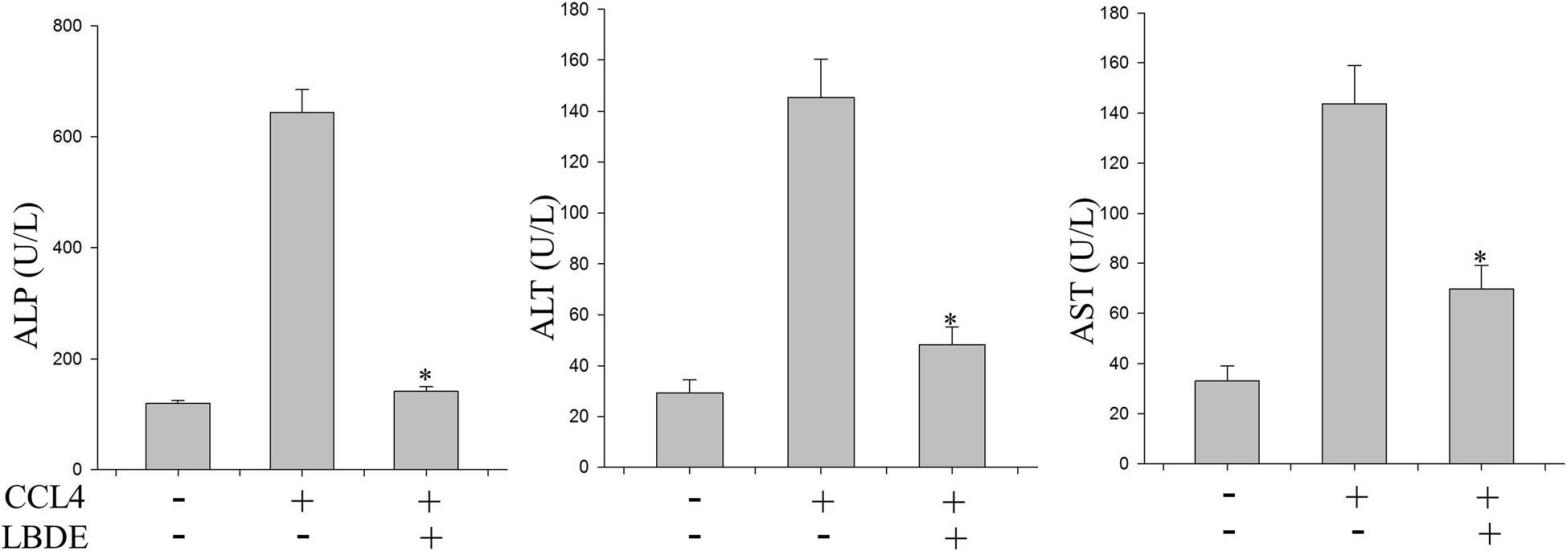
Analysis of ALP, AST and ALT levels in serum after treatment with CCL-4 and LBDE

To confirm the functions of the liver, activities of alkaline phosphatase (ALP), aspartate aminotransferase (AST) and alanine aminotransferase (ALT) were assayed. As shown in Fig. 4 levels of ALT, AST and ALP were increased to three-fold when treated with CCl_4_ and reduced back to normal range when treated with LBDE.

### LBDE treatment improved CCL4-induced liver damage

**Fig. 5:**
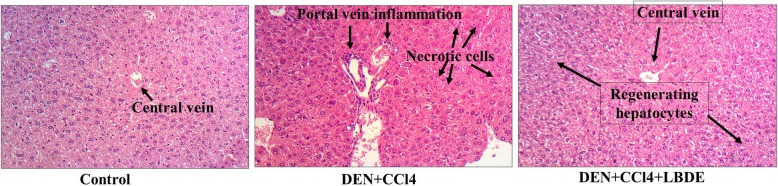
Light microscopy showing H & E staining of normal liver tissue in control group, degenerated and necrotic liver cells associated with inflammatory cells in CCL-4 treated group and the pathological change of liver as observed after treatment with LBDE

Histopathology of liver tissues was performed for morphological examination. This was done by H&E staining and results were compared among all experimental groups, control, CCl_4_ treated and CCl_4_ + LBDE treated. As shown in Fig. 5, liver cells of the control group had normal architecture and with no marked inflammatory signs, while hepatocytes of CCl_4_ treated group of mice were highly dysplastic and the presence of inflammatory cells was observed mainly around the portal tract. LBDE treatment reversed the inflammatory and dysplastic conditions of liver cells as shown in Fig. 5. LBDE treated mice group hepatocytes showed normal architecture with minimum signs of inflammation and dysplasia.

### LBDE treatment suppressed NF-κB and COX-2 activation in-vitro and in-vivo

**Fig. 6:**
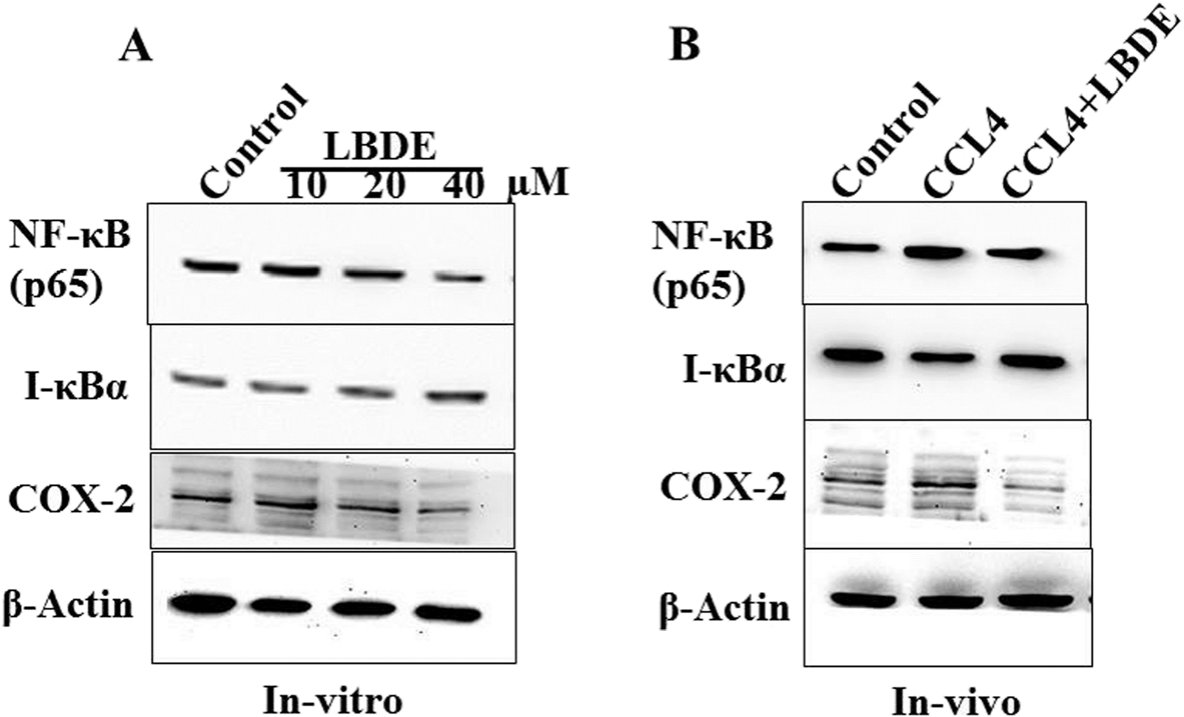
LBDE inhibited NF-κB and COX-2 in CCL-4-induced hepatic fibrosis. **a** HepG2 cells were exposed to various concentration of LBDE, after which total protein content was isolated from cells and analysed by Western blotting with various antibodies. **b** Developed tumors in flanks of mice were cut-out, and tissues were homogenized with tissue lysis buffer. Total protein content was isolated from cells and analysed by Western blotting. Actin was used as a loading control in both experiments

NF-кB and COX-2 are key inflammatory regulators and elevated expressions have been noted in several inflammatory conditions. In this study, expression of NF-κB, I-κB and COX-2 proteins were analysed with various doses of LBDE against HepG2 cell lines. LBDE decreased the expression of both NF-κB and COX-2 inflammatory regulators at 40 μM as well as increased the expression of I-κB, a negative regulator of NF-κB (Fig. 6a). CCl_4_ significantly increased the NF-κB and COX-2 expression advocating inflammatory condition and cell dysplasia. CCl_4_ treatment also decreased the expression of I-κB. LBDE treatment not only decreased the expression of NF-κB and COX-2, but also restored the expression of I-κB in liver tissue (Fig. 6b). Actin was used as a loading control in western blot experiments.

## Discussion

Inflammation is a desirable natural protective mechanism of the immune system against a wide range of noxious and harmful stimuli such as pathogens, toxins, irradiation and trauma, toxic chemicals and minerals and antigens [2, 8]. During the inflammatory process, cellular and molecular events take place, which rapidly changes the vasculature and combat with threatening injuries or infections. These microcirculatory changes including vascular permeability and inflammatory mediators restore tissue homeostasis as well as cause resolution of the acute inflammatory response [28]. However, uncontrolled and persistent acute inflammation may change to a chronic process, causing a variety of chronic inflammatory diseases such as cardiovascular, hepatic, metabolic, and cancers [28]. Chronic inflammation is associated with the infiltration of Kupffer cells and HSCs and releases pro-inflammatory cytokines causing hepatocyte damage and hepatic diseases [29, 30]. Our study showed that treatment with a natural lignin LBDE decreased cell viability and expression of inflammatory mediators NF-κB and COX-2 as well as serum levels of ALT, AST, and ALP in a CCl4-induced hepatic fibrotic model. The morphological examination also confirmed that LBDE treatment reduced the infiltration of inflammatory cells and improved liver necrosis with no observed pseudoloculus compared to the CCl4 group. These results demonstrated that LBDE significantly protected the liver from CCl4 toxicity, which can be correlated with its inhibitory effect against inflammation.

CCl4 has been used as a model carcinogen for the establishment of the hepatic fibrotic experimental model because it displays similar hepatic pathology in animals as well as in humans [31]. Studies have shown that the administration of CCl4 increased inflammatory cytokines, disrupted immune system and lymphatic organs, subsequently resulted in chronic inflammatory hepatic fibrosis [32, 33]. Additionally, CCl4 increased the levels of ALP, AST and ALT in the serum reflecting the degree of hepatic fibrosis. During hepatic fibrosis, these enzymes are released into the circulation from cytoplasm and mitochondria, and their levels determined the functionality of the liver [34]. Our results show that the ALP, AST and ALT levels were much higher in the CCl4 treated groups consistent with the previously reported studies. However, LBDE treatment decreased the levels of hepatobiliary enzymes indicating that LBDE ameliorated CCl4-induced liver injury and improved liver function.

Previous studies have shown NF-κB as a master regulator of inflammation by producing proinflammatory cytokines in the liver, activating HSCs subsequently causing HCC and hepatic diseases [35]. Out of five, p50 and p65 heterodimers are involved in gene transcription and cancer development. In most cells, NF-κB is sequestered by inhibitory protein I-κBin the cytoplasm. I-κB inhibits NF-κBp65 from nuclear translocation and transcription of inflammatory genes [35]. But in case of liver injury or inflammation, I-κB is phosphorylated and degraded, thus resealing NF-κB where it activates downstream targets to regulate inflammatory gene transcription involved in HSC activation [36]. NF-κB activation has been noted in the HCC model of mice when injected with the carcinogens CCl4 and DEN [35, 37]. CCl4 and DEN require assistance from concurrent inflammation followed by NF-κB activation in Kupffer cells and liver macrophages [35, 37]. It is a well-known fact that inhibition of NF-κB and NF-κB activated downstream targets in Kupffer cells reduces the development of HCC. Our results are in agreement with previously reported studies that CCl4 and DEN activated NF-κBp65. However, LBDE treatment significantly modulated CCl4-induced NF-κB activation possible by preventing I-κBα phosphorylation and degradation.

Among various NF-κB target genes, COX-2 is the best known for the promotion and development of inflammatory conditions including HCC and hepatic fibrosis [38]. COX-2 is an inflammatory molecule, which is induced by various stimulus including cellular stress, inflammatory, and carcinogens, subsequently releasing prostaglandin (PG) promoting tumor development and metastasis [39]. The liver function has been improved by inhibiting NF-κB induced-activation of proinflammatory factors such as tumor necrosis factor-alpha (TNFα), interleukin-6 (IL-6), and PGE2, and iNOS, COX-2, and matrix metallopeptidase-9 in LPS-stimulated RAW 264.7 macrophages as well as in acute liver fibrosis mouse model [38, 40, 41]. Lignan can inhibit binding affinity between NF-κB and AP-1 transcription factors to interleukins (IL-6 promoter and IL-6) production through p38/NF-κB and AP-1 signaling pathways in cancer [42]. It has been reported that LBDE repressed the activation of COX-2 and iNOS genes by blocking the activation of NF-κB [12, 15, 23]. Therefore, blocking the activation of NF-κB and COX-2 could prevent the recruitment of inflammatory mediators in hepatic diseases. LBDE decreased CCl4-induced activation of COX-2, consequently reduced inflammation, necrosis, and fibrosis. Taken together, our results suggest that LBDE attenuates inflammation in the liver, at least in part, by suppressing the NF-κB and COX-2 signalling pathway.

## Conclusion

In conclusion, our results demonstrated that NF-κB and COX-2 are responsible for the inflammation-induced development of hepatic fibrosis. Therefore, inhibition of the NF-κB/COX-2 pathway may provide a prospective strategy for the prevention and treatment of hepatic diseases. This preliminary study highlighted the therapeutic potential of natural compound LBDE in HCC and hepatic fibrosis. LBDE improved hepatobilliary enzymes and downregulation of NF-κB and COX-2 in mice model of hepatic fibrosis. Since hepatic fibrogenesis is a very complex condition process, the detail underlying mechanisms of LBDE action remain to be investigated.

## Supplementary information

**Additional file 1.** SI values calculation file.

## Data Availability

All data are presented in the manuscript. Further results and analysis data are available with the corresponding author on reasonable request.

## Abbreviations

ALP: Alkaline Phosphatase
ALT: Alanine Aminotransferase
AST: Aspartate aminotransferase
CCl_4_: Carbon Tetrachloride
COX-2: Cyclooxygenase-2
DEN: Diethylnitrosamine
HCC: Hepatocellular carcinoma
iNOS: inducible nitric oxide synthase
IκBα: I kappa B alpha
LBDE: Lirioresinol B Dimethyl Ether
MMP-9: Matrix metallopeptidase 9
NFκB: Nuclear factor kappa B
PGE_2_: Prostaglandin E_2_
PTHrP: Parathyroid hormone-related protein
